# Seasonal Variation in *TP53 R249S*-Mutated Serum DNA with Aflatoxin Exposure and Hepatitis B Virus Infection

**DOI:** 10.1289/ehp.1103539

**Published:** 2011-07-18

**Authors:** Stéphanie Villar, Emilie Le Roux-Goglin, Doriane A. Gouas, Amelie Plymoth, Gilles Ferro, Mathieu Boniol, Myriam Lereau, Ebrima Bah, Andrew J. Hall, Christopher P. Wild, Maimuna Mendy, Helene Norder, Marianne van der Sande, Hilton Whittle, Marlin D. Friesen, John D. Groopman, Pierre Hainaut

**Affiliations:** 1 Molecular Carcinogenesis Group, International Agency for Research on Cancer, Lyon, France; 2 Gambia Hepatitis Intervention Study, Laboratories Fajara, Banjul, the Gambia; 3 Department of Infectious Disease Epidemiology, London School of Hygiene and Tropical Medicine, London, United Kingdom; 4 Medical Research Council, Laboratories Fajara, Banjul, the Gambia; 5 Swedish Institute for Infectious Disease Control, Solna, Sweden; 6 National Institute for Public Health and the Environment, Bilthoven, the Netherlands; 7 Johns Hopkins University Bloomberg School of Public Health, Baltimore, Maryland, USA

**Keywords:** 1762^T^/1764^A^, circulating DNA, mycotoxin, seasonality, *TP53* mutation

## Abstract

Background: Chronic hepatitis B virus (HBV) infection and dietary aflatoxin B_1_ (AFB_1_) exposure are etiological factors for hepatocellular carcinoma (HCC) in countries with hot, humid climates. HCC often harbors a *TP53* (tumor protein p53) mutation at codon 249 (*R249S*). In chronic carriers, 1762^T^/1764^A^ mutations in the HBV *X* gene are associated with increased HCC risk. Both mutations have been detected in circulating cell-free DNA (CFDNA) from asymptomatic HBV carriers.

Objective: We evaluated seasonal variation in *R249S* and HBV in relation to AFB_1_ exposure.

Methods: *R249S* was quantitated by mass spectrometry in CFDNA in a cross-sectional survey of 473 asymptomatic subjects (237 HBV carriers and 236 noncarriers) recruited in three villages in the Gambia over a 10-month period. 1762^T^/1764^A^ HBV mutations were detected by quantitative polymerase chain reaction. In addition, the HBV *S* gene was sequenced in 99 subjects positive for HBV surface antigen (HBsAg).

Results: We observed a seasonal variation of serum *R249S* levels. Positivity for *R249S* and average concentration were significantly higher in HBsAg-positive subjects surveyed during April–July (61%; 5,690 ± 11,300 *R249S* copies/mL serum) than in those surveyed October–March [32% and 480 ± 1,030 copies/mL serum (odds ratio = 3.59; 95% confidence interval: 2.05, 6.30; *p* < 0.001)]. Positivity for HBV e antigen (HBeAg) (a marker of HBV replication) and viral DNA load also varied seasonally, with 15–30% of subjects surveyed between April and June HBeAg positive, compared with < 10% surveyed during other months. We detected 1762^T^/1764^A^ mutations in 8% of carriers, half of whom were positive for *R249S*. We found HBV genotype E in 95 of 99 HBsAg-positive subjects.

Conclusion: *R249S* is detectable in CFDNA of asymptomatic subjects. Evidence of temporal and quantitative variations suggests an interaction among AFB_1_ exposure, HBV positivity, and replication on *TP53* mutation formation or persistence.

About 80% of the 700,000 estimated annual cases of hepatocellular carcinoma (HCC) occur in low-resource, intertropical areas, mainly in sub-Saharan Africa and Southeast Asia (McGlynn and London 2005; Sherman 2005). The two major risk factors, chronic hepatitis B virus (HBV) infection and exposure to aflatoxin B_1_ (AFB_1_), have been classified as group 1 carcinogens by the International Agency for Research on Cancer (IARC 1994, 2002), with a synergistic effect on HCC risk (Kew 2003). Both factors are preventable in principle (Wild and Hall 2000). The presence of a specific AGG → AGT mutation induced by AFB_1_ at codon 249 of *TP53* tumor suppressor gene (*R249S*) is observed in > 50% of HCC in geographic areas with high incidence. Worldwide, there is a remarkable ecological correlation among AFB_1_ exposure, incidence of HCC, and prevalence of the *TP53 R249S* mutation in HCC tumors (Gouas et al. 2009). This mutation occurs early in the pathway leading to HCC and may thus provide an early biomarker of AFB_1_ exposure and hepatocarcinogenesis (Jackson et al. 2003).

Several studies have shown that plasma or serum of cancer patients contains significant amounts of circulating cell-free DNA (CFDNA), which often carries mutations and epigenetic alterations identical to those detected in tumor tissues (Gormally et al. 2007). Although the mechanisms of release and the stability of mutant DNA in the bloodstream are still unclear, CFDNA may serve as a convenient biomarker in cancer subjects. Importantly, traces of free fragments of DNA are also detectable in most healthy subjects (Gormally et al. 2007). In several instances, this DNA has been found to harbor mutations that predict cancer occurrence (Gormally et al. 2006; Jackson et al. 2003).

Double mutations in the HBV *X* gene (adenine to thymine, 1762^T^; and guanine to adenine, 1764^A^) have been observed in HCC and liver cirrhosis (Arbuthnot and Kew 2001; Baptista et al. 1999; Hou et al. 1999). This mutation is also detectable in CFDNA up to 5 years before HCC diagnosis (Kuang et al. 2004; Yuan et al. 2009). HCC risk is increased in chronic carriers with 1762^T^/1764^A^ mutations, and it increases with higher serum concentrations of 1762^T^/1764^A^ (Yuan et al. 2009).

In prospective studies in China, *R249S* was detected in the plasma of asymptomatic HBV carriers up to several years before HCC diagnosis (Jackson et al. 2003). In a case–control study in the Gambia, West Africa, we used a quantitative method to detect the *R249S* mutation by electrospray mass spectrometry [short oligonucleotide mass analysis (SOMA)] (Lleonart et al. 2005). Plasma concentrations of *R249S* were increased in HCC cases (median, 2,800 copies/mL; range, 500–11,000 copies/mL) compared with controls (median, 500 copies/mL; range, 250–2,000 copies/mL). However, low levels of *R249S* (> 500 copies/mL) were detected in 46% of asymptomatic controls, which suggests that the mutation may also serve as a marker of ongoing exposure to aflatoxin (Lleonart et al. 2005).

In the Gambia, chronic HBV infection is endemic, affecting 11–16% of the adult population (Kirk et al. 2006). AFB_1_ exposure also is widespread, and the annual incidence of HCC peaks at 80 cases per 100,000 among 35- to 55-year-old males (Bah et al. 2001). *R249S* was detectable in the plasma of 40% of HCC patients, with a concordance of 88% between plasma and tumor DNA (Szymañska et al. 2004). *R249S* was also found in 15% of patients with liver cirrhosis and in 3% of control subjects with no detected liver disease (Kirk et al. 2005). Furthermore, the 1762^T^/1764^A^ HBV mutation was found in 48% (10 of 21) of asymptomatic carriers, 75.4% (86 of 114) of HCC patients, and 77% (33 of 43) of patients with cirrhosis (Mendy et al. 2008). This mutation has also been found in the serum of 66% of HCC patients and 11% of asymptomatic carriers from South Africa (Baptista et al. 1999). These observations led us to hypothesize that circulating *R249S* may be a biomarker of early liver disease or, alternatively, a biomarker of ongoing AFB_1_ exposure, and to further assess the prevalence of 1762^T^/1764^A^ mutation in asymptomatic subjects from the Gambia who are positive for HBV surface antigen (HBsAg). Therefore, we evaluated seasonal variation in *R249S* and HBV in relation to AFB_1_ exposure.

## Materials and Methods

*Study participants.* A total of 473 asymptomatic study participants were recruited from the population of three rural villages in the Gambia (Keneba, Manduar, and Katong-Kunda) where exposure to aflatoxin is widespread and well documented; a previous study in Keneba in the Gambia showed a correlation between measurement of dietary intake of aflatoxin and levels of albumin-bound aflatoxin in serum (Wild et al. 1992). These villages are part of a long-term quadrennial survey program for HBV infection initiated in the early 1980s as a pilot and sentinel program for the Gambian Hepatitis Intervention Study (Whittle et al. 2002). In this rural population, dietary exposure is widespread because of the use of groundnuts as the main basis of diet, whereas exposure to other HCC risk factors such as alcohol is negligible. We used a series of serum specimens (*n* = 491) collected for the survey from October 2002 to July 2003 (van der Sande et al. 2006) to assemble a case–control study including 237 HBsAg-positive (HBV carriers) and 236 HBsAg-negative (noncarriers) individuals matched by date of sampling, age, and sex. Previous studies have shown that serum or plasma are both suitable sources of CFDNA (reviewed by Gormally et al. 2007). None of the participants had a clinical diagnosis of chronic liver disease at the time of sampling, although no detailed clinical assessment of their liver status was conducted. All participants gave informed consent for inclusion in the quadrennial survey. The present protocol was reviewed and approved by the ethical review boards of the Medical Research Council in the Gambia and of IARC. This study complied with all international standards.

*HBV serology.* We tested subjects for HBV core antibodies (anti-HBc) and, if positive, HBsAg and HBV e antigens (HBeAg). Anti-HBc was measured using the radioimmunoassay AB-COREK test kit (Sorin Biochemica, Saluggia, Italy) according to the manufacturer’s protocol. Anti-HBc positive samples were tested for HBsAg by reverse passive hemagglutination assay (Wellcotest_®_; Murex Diagnostics, London, UK) and/or by the Determine™ HBsAg immunochromatographic assay (Abbott Laboratories, Abbott Park, IL, USA). HBsAg-positive subjects were tested for HBeAg using an enzyme immunoassay (Equipar Diagnostici, Rome, Italy).

*CFDNA extraction and quantitation.* CFDNA was extracted from 300 μL serum using the QIAmp DNA Blood Mini Kit (Qiagen, Hilden, Germany) according to the protocol described previously (Szymañska et al. 2004). DNA was eluted in 200 μL elution buffer. Quantitation of extracted CFDNA was performed by fluorimetry using PicoGreen (Interchim, Montluçon, France).

*Quantitation of* R249S *in CFDNA. R249S* was quantitated by SOMA as described previously (Lleonart et al. 2005), with the following modifications. Before polymerase chain reaction (PCR) amplification of a 93-base-pair segment of exon 7 of *TP53* encompassing codon 249, 228 copies of an internal standard plasmid were added to all DNA extracts to provide a reference for quantitation. After restriction digestion with *Hae*III, which specifically cuts the wild-type sequence of codon 249, the mutated and internal-standard–enriched PCR products were reamplified, cut with *GsuI* to produce short 8-mer oligonucleotides, purified, and quantitated by HPLC/electrospray ionization mass spectrometry. Results were expressed as concentrations (copies of *R249S* per milliliter of serum). Levels ranged from nondetectable to 63,800 copies/mL. The elimination of the wild-type sequence of codon 249 by restriction digestion increased the sensitivity of the method relative to the original method described by Lleonart et al. (2005). Consequently, the limit of detection was 70 copies/mL serum. Samples with serum concentrations > 70 copies/mL were classified as positive for *R249S*.

*HBV DNA quantitation and 1762^T^/1764^A^ analysis.* We performed HBV DNA quantitation as described previously (Mendy et al. 2006). 1762^T^/1764^A^ mutation analysis was performed by quantitative PCR (Yuan et al. 2009).

S *gene sequencing.* Analysis of the *S* gene provides genotype information that significantly matches analysis of the entire genome (Hussain et al. 2003; Norder et al. 1993). We developed a new semi-nested PCR amplifying the entire *S* gene. The first reaction was achieved on 5 μL DNA with primers S_HBVpol1 (5´-cctgctggtggctccagttca-3´) and S_HBVporv2 (5´-aaagcccaaaagacccacaat-3´); round settings were 95°C (15 min); 40 cycles of 95°C (30 sec), 60°C (30 sec), 72°C (1 min); and then 72°C for 7 min. The second step used 1 μL first reaction product and primers S_HBV123s (5´-tcgaggattggggaccctg-3´) and S_HBVporv2; round settings were 95°C (15 min); 45 cycles of 95°C (30 sec), 58°C (30 sec), 72°C (1 min); and then 72°C for 7 min. PCR products (5 μL) were purified using standard ExoSap-IT_®_ enzyme, (USB_®_ Corporation, Cleveland, OH, USA) and nucleotide sequences were determined for both strands by automated dideoxy sequencing (AbiPrism 3100 sequencer; Applied Biosystems, Carlsbad, CA, USA). Direct sequencing on amplified fragments was performed using the primers S_HBV123s, S_HBVporv2, and S_HBV778r (5´-gaggtataaagggactcaag-3´). HBV genotypes and subtypes were determined in collaboration with the Virological Department of Swedish Institute for Infectious Disease.

*Statistical analysis.* Values were expressed in numbers of *R249S* copies/mL serum, with resulting values between 0 and 63,800 copies/mL. For statistical analyses, values below the detection limit of 70 copies/mL were assigned a value of 70 copies/mL. Results showed large variations and were analyzed in three ways. First, we evaluated the distribution of values according to month of collection. Second, means ± SDs were calculated for groups of months corresponding to two periods of different levels of exposure to aflatoxin. Third, the number and percentage of subjects with values > 70 copies/mL were tabulated in relation with HBV serological status or the two periods of different aflatoxin exposure. To analyze the impact of seasonal AFB_1_ exposure, we grouped results into two seasons: *a*) a higher AFB_1_ exposure period from October 2002 to March 2003, after the September–October harvest and including the dry season in February–March; and *b*) a lower AFB_1_ exposure period from April 2003 to July 2003, which included part of the June–August wet season. AFB_1_ accumulates in foodstuffs (principally groundnuts in the Gambia) after harvest in September–October; levels of AFB_1_–albumin adducts (a biomarker of recent AFB_1_ exposure) have been shown to decrease from their highest levels in the February–March dry season to reach their lowest levels after the June–August wet season just before the next harvest (Turner et al. 2000; Wild et al. 2000). Statistical analyses were performed using STATA software (version 11.1; StataCorp LP, College Station, TX, USA) or by Student’s *t*-test to compare the average of *R249S* copies per milliliter in HBsAg-positive or -negative subjects depending on AFB_1_ exposure. Analysis involved standard unconditional logistic regression of the probability of *R249S* positivity using as explanatory variables HBsAg status and seasonality adjusted for sex and age.

## Results

The characteristics of study participants are described in Table 1. Most individuals were recruited in Keneba and Manduar villages (HBsAg positive, 38% and 40%, respectively; HBsAg-negative, 52% and 28%). Almost all HBsAg-positive subjects had not been vaccinated (92%), whereas 44% of HBsAg-negative subjects had been vaccinated. Mean serum CFDNA concentrations (± SD) were not significantly different (*p* < 0.73) for HBsAg-positive subjects (0.11 ± 0.92 ng/μL) compared with HBsAg-negative subjects (0.09 ± 0.51 ng/μL).

**Table 1:** Descriptive characteristics of participants in the study grouped by HBsAg status.

Characteristic	HBsAg positive	HBsAg negative
No. of subjects		237		236
Mean age (range), years		31 (5–69)		31 (5–70)
Sex [*n* (%)]				
Male		109 (46)		108 (46)
Female		128 (54)		128 (54)
Marker of viral replication [male/female,* n* (%)]				
HBeAg positive		17/10 (16/8)		—
HBeAg negative		92/118 (84/92)		—
HBV vaccination [*n* (%)]				
Unknown		2 (1)		—
Yes		17 (7)		104 (44)
No		218 (92)		132 (56)
Village [*n* (%)]				
Keneba		89 (38)		123 (52)
Manduar		95 (40)		66 (28)
Katong-Kunda		41 (17)		36 (15)
Other		12 (5)		11 (5)

A total of 164 subjects (106 HBsAg positive and 58 HBsAg negative) had *R249S* serum concentrations > 70 copies/mL and were considered positive for *R249S* (Table 2). HBsAg-positive subjects were more likely to be *R249S* positive than HBsAg-negative subjects [45% vs. 25%, respectively; odds ratio (OR) = 2.49; 95% confidence interval (CI): 1.68, 3.69].

**Table 2:** Subjects positive for serum *R249S* mutation by season [*n*/total (%)].

Period	HBsAg negative*a*	HBsAg positive	OR (95% CI)
October–July		58/236 (25)		106/237 (45)		2.49 (1.68, 3.69)*b*
October–March (higher AFB_1_ exposure)		45/134 (34)		43/134 (32)		0.94 (0.56, 1.57)
April–July (lower AFB_1_ exposure)		13/102 (13)		63/103 (61)		10.9 (5.35, 22.34)
ORs are adjusted for age and sex. **a**Used as reference category for OR determination. **b**Adjusted for seasonality.						

**Figure 1:**
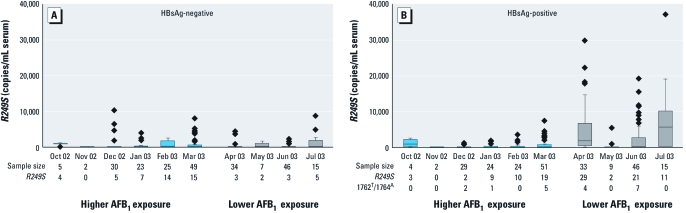
Box and whisker plots for the distribution of serum concentrations of *TP53* *R249S* in HBsAg-negative (*A*) and -positive (*B*) subjects during higher and lower AFB_1_ exposure periods. Boxes extend from the 25th to the 75th percentile; horizontal lines within the boxes represent the median; whiskers extend 1.5 times the length of the interquartile range above and below the 75th and 25th percentiles, respectively; and diamonds represent outliers. Four outliers > 40,000 copies/mL serum are not represented: one in March for HBsAg-negative subjects, and one in April and two in June for HBsAg-positive subjects. The number of individuals sampled (2–51 subjects/month) and the distribution of HBsAg-positive subjects also positive for the 1762^T^/1764^A^ double mutation are given.

Serum *R249S* concentrations were low for both HBsAg-negative and -positive subjects during the higher AFB_1_ exposure period (October–March) (Figure 1). However, during the lower AFB_1_ exposure period (April–July), both serum concentrations and the number of subjects positive for *R249S* appeared to increase in the HBsAg-positive group.

**Figure 2:**
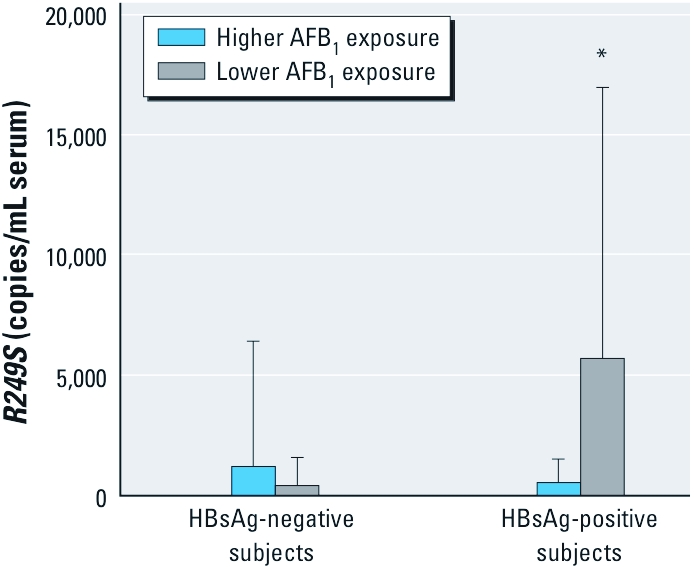
Serum concentrations (mean ± SD) of *TP53* *R249S* in HBsAg-positive and ‑negative subjects, grouped by seasonal AFB_1_ exposure. *p < 0.001 compared with HBsAg-negative subjects with lower AFB_1_ exposure, and compared with HBsAg-positive subjects with higher AFB_1_ exposure, by Student’s *t*-test.

Mean (± SD) serum concentrations of *R249S* were similar for HBsAg-negative subjects during the higher (1,183 ± 5,230 copies/mL serum) and lower (400 ± 1,160 copies/mL serum) AFB_1_ exposure periods (*p* = 0.137) and for HBsAg-positive (1,183 ± 5,230 copies/mL serum) and HBsAg-negative (480 ± 1,030 copies/mL serum) subjects (*p* = 0.128) during the higher AFB_1_ exposure period (Figure 2). In contrast, the mean serum concentration of *R249S* in HBsAg-positive subjects during the lower AFB_1_ exposure period (5,690 ± 11,300 copies/mL serum) was significantly higher than in HBsAg-negative subjects during the same period (*p* < 0.001) and HBsAg-positive subjects during the higher AFB_1_ exposure period (*p* < 0.001). We also observed this seasonal variation when the data were analyzed qualitatively (Table 2). The proportion of *R249S*-positive samples was highest among HBsAg-positive subjects during the lower AFB_1_ exposure period (April–July) and lowest among HBsAg-negative subjects during the same period (61% and 13%, respectively; OR = 10.9; 95% CI: 5.35, 22.34) (Table 2). In contrast, the proportions of *R249S*-positive samples were comparable between HBsAg-positive and -negative subjects during the higher AFB_1_ exposure period (October–March; 32% and 34%, respectively; OR = 0.94; 95% CI: 0.56, 1.57). Thus, in HBsAg-positive subjects both the levels of *R249S* in serum (Figure 2) and *R249S* positivity (Table 2) peaked several months after the previously reported high AFB_1_ exposure period in January–February. In HBsAg-positive subjects, 61% were *R249S* positive during the lower AFB_1_ exposure period compared with 32% *R249S* positive during the higher AFB_1_ exposure period (OR = 3.59; 95% CI: 2.05, 6.30) (Table 3).

**Table 3:** Subjects positive for serum *R249S* mutation by HBsAg status [*n*/total (%)].

HBsAg	October–March*a*	April–July	OR (95% CI)
Negative		45/134 (34)		13/102 (13)		0.30 (0.15, 0.61)
Positive		43/134 (32)		63/103 (61)		3.59 (2.05, 6.30)
ORs are adjusted for age and sex. **a**Used as reference category for OR determination.						

Among HBsAg-positive subjects, 45% were *R249S* positive, 8% were positive for the 1762^T^/1764^A^ HBV mutation, and 4% were positive for both *R249S* and 1762^T^/1764^A^ (Table 4). Subjects who had both *R249S* and 1762^T^/1764^A^ were an average of 5–11 years younger than other subjects [mean age (range) 26 (7–49) years, compared with 31 (7–65) years for *R249S* positive only, 37 (20–61) years for 1762^T^/1764^A^ positive only, and 31 (5–68) years for negative for both mutations]; however, this difference was not statistically significant (*p* = 0.08). Serum *R249S* positivity did not vary significantly according to 1762^T^/1764^A^ status (Table 4), village of origin, sex, vaccination status, HBe status, or serum concentration of viral DNA (data not shown).

**Table 4:** HBsAg-positive subjects positive for serum *R249S* mutation and/or 1762^T^/1764^A^ double mutation [*n* (%)].

1762^T^/1764^A^ mutation		
*R249S* mutation status	No	Yes	Total
No		122 (51)		9 (4)		131 (55)
Yes		96 (41)		10 (4)		106 (45)
Total		218 (92)		19 (8)		237 (100)
For 1762^T^/1764^A^ compared with *R249S* mutation status, χ^2^ = 0.5222; *p* = 0.470.						

**Figure 3:**
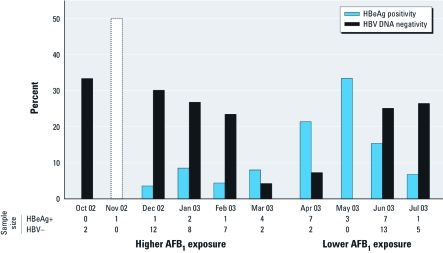
Seasonal variation of HBeAg positivity (HBeAg+) and HBV DNA negativity (HBV–) in HBsAg-positive subjects. The number of individuals sampled each month are given. The value for November 2002 was not considered because the sample size was too small.

HBeAg status and presence of detectable HBV DNA also showed seasonal variation. Among HBsAg-positive subjects, the proportion that were HBeAg positive (indicating active viral replication) was highest during April–June, whereas the proportion with undetectable HBV DNA decreased to < 10% between March and May, consistent with a seasonal peak in viral replication at the transition during high and low AFB_1_ exposure periods, slightly ahead of the observed peak of *R249S* DNA (Figure 3). The seasonal patterns of *R249S*, HBeAg, and HBV DNA were not associated with obvious differences in total serum CFDNA concentrations (data not shown).

To gain insight into the viral factors that may affect the occurrence of *R249S* in serum, we sequenced the entire *S* gene of HBV DNA. A close correlation exists between serological subtypes and genotypes of HBV (Kramvis et al. 2005; Norder et al. 1992, 2004), and the sequence coding for HBsAg can be used for genotyping. We amplified the serum DNA of all HBsAg-positive subjects by PCR and obtained nonambiguous sequences for 99 subjects, 51 of whom were positive for *R249S*. Among these sequences, 95 were of genotype E and 4 were of genotype A [see Supplemental Material, Figure S1 (http://dx.doi.org/10.1289/ehp.1103539)]. Genotype E sequences were diverse and did not represent a particular cluster within the current phylogenetic tree of this genotype. A total of 15 individuals sequenced for *S* gene were carriers of the 1762^T^/1764^A^ double mutation. To further determine whether the presence or level of *R249S* may vary according to particular subgroups, we subdivided subjects with sequences belonging to genotype E into five groups based on sequence similarities. Three distinct *S* gene sequences were found: one in 8 individuals (group 1), another in 17 individuals (group 2), and the third in 16 individuals (group 3). Group 4 comprised 12 subjects with closely related sequences, and group 5 comprised all other subjects (42) with unique *S* gene sequences. We found no significant difference between levels of *R249S* or presence of 1762^T^/1764^A^ double mutation and *S* sequence groups after adjustment for seasonality (data not shown).

## Discussion

We used a sensitive and quantitative method to detect *TP53 R249S* in the serum of individuals from a rural area of the Gambia with no evidence of chronic liver disease. We obtained serum samples in a cross-sectional survey performed in 2002–2003 in three villages where consumption of AFB_1_-contaminated foodstuff and individual variations in exposure to AFB_1_ have been previously documented (Wild et al. 2000). Two matched groups of subjects were tested: subjects positive for HBsAg (chronic HBV carriers) and control subjects seronegative for HBsAg. We detected *R249S* more frequently in HBV carriers (45%) than in controls (25%; OR = 2.49; 95% CI: 1.68, 3.69). In both groups, we observed seasonal variations in the proportion of subjects positive for *R249S* and in the average concentration of serum *R249S* DNA. Our results showed that *a*) *R249S* was detectable in CFDNA of a significant proportion of subjects with no symptoms of chronic liver disease; *b*) *R249S* positivity was associated with HBV carrier status; and *c*) the presence and levels of *R249S* in CFDNA varied according to season.

In the Gambia, groundnut culture occupies > 50% of harvested land (Department of Agricultural Services, the Gambia 2008). Groundnuts are the main source of exposure to aflatoxins and are consumed seasonally according to availability. AFB_1_ accumulates in foodstuffs after harvest in September–October and peaks in January–February, when the availability of stored groundnuts and the relative lack of other crops lead to significantly higher exposure than during other months. We did not measure aflatoxin exposure in the present study, but the seasonality of exposure is well documented in adults (Wild et al. 1992) and children (Turner et al. 2000) in the Gambia. In addition, Wild et al. (2000) reported seasonal variation in AFB_1_–albumin adduct levels in a longitudinal study of residents in the same villages included in the present study, with the highest level in February–March (83.2 pg AFB_1_-lysine/mg albumin; 95% CI: 53.3, 130.8) and the lowest levels in June–July (34.9 pg AFB_1_-lysine/mg albumin; 95% CI: 28.5, 42.8). In nonvaccinated children 3–4 years of age from four geographical areas representative of the Gambia as a whole, AFB_1_–albumin adducts were detected in all samples (range, 2.2–459 pg AFB_1_-lys lysine/mg albumin), and levels were twice as high in November–March as in May–October (monthly mean levels, 75.7–88.7 and 27.8–50.3 pg AFB_1_-lys lysine/mg albumin, respectively). Adduct levels were significantly higher in acutely and chronically HBV-infected children, suggesting that infection may modulate AFB_1_ metabolism and DNA adduct formation (Wild et al. 2000).

In the present study, we took this research one step further by showing evidence of seasonal variation in the *R249S TP53* mutation in CFDNA. This mutation is a consequence of the formation of AFB_1_–DNA adducts (Besaratinia et al. 2009; Gouas et al. 2009). In HBV-negative subjects, temporal patterns were similar to those described for AFB_1_–albumin adducts, with a lower proportion positive for *R249S* (13%) among those surveyed during April–July (lower AFB_1_ exposure period) than during October–March (34%; higher AFB_1_ exposure period; OR = 0.30; 95% CI: 0.15, 0.61). In contrast, we observed the opposite pattern in HBV carriers, with a greater proportion positive for *R249S* (61%) among those surveyed during April–July than during October–March (32%; OR = 3.59; 95% CI: 2.05, 6.30). Furthermore, the average concentration of *R249S* in serum was higher in subjects surveyed in April–July than in those sampled in October–March (*p* < 0.001). The process of adduction and fixation of mutation and persistence of the mutation require several steps, which could partly explain the apparent lag between increased detection of AFB_1_–albumin adducts (a short-term biomarker of exposure to aflatoxin) and increased detection of *R249S* in CFDNA (which may occur days or weeks after exposure). However, taking these steps into account does not explain the observed difference between controls and HBsAg-positive subjects. Therefore, our results suggest that the dynamics and persistence of *R249S* mutations may be different in HBV carriers than in controls, consistent with an interaction between chronic HBV infection and *R249S* formation, amplification, and persistence.

Positivity for HBeAg and presence of detectable HBV DNA in the serum also showed seasonal variations, with a peak at the transition during periods of high and low AFB_1_ exposure. Seasonal reactivation of HBV might induce damage to hepatocytes and increase *R249S* release into the bloodstream in the weeks that follow reactivation. Seasonal hepatitis flare was described by Zhang et al. (2006) in a longitudinal study of 2,238 subjects from the area of Guangzhou, China. In that study, acute exacerbation of hepatitis peaked during spring (March–May), and HBeAg seroconversion peaked during summer (June–August). So far, there is no report on seasonal patterns of HBV infection in West Africa.

To gain further insights on the role of HBV, we analyzed double mutations in the HBV *X* gene (1762^T^/1764^A^) that have been associated with infection severity. A recent prospective study in Thailand showed that chronic HBV carriers with 1762^T^/1764^A^ double mutations had an elevated risk of HCC (OR = 2.47; 95% CI: 1.04, 5.85) (Kuang et al. 2005). In the Gambia, we observed this double mutation in some chronic carriers who were asymptomatic for chronic liver disease, and in most HCC or liver cirrhosis cases (Mendy et al. 2008). In the present series, we found 1762^T^/1764^A^ double mutations in 19 of 237 HBV carriers (8%), with no association with *R249S* positivity. Sequencing of the entire *S* gene in 99 carriers detected genotype E in 95 cases and genotype A in four cases. We found no association between subgroups of genotype E and *R249S* positivity.

Although there are no data on the stability of *R249S* in serum, its presence at detectable levels may require the release of DNA from a large number of cells harboring mutant DNA. The limited data available on serum DNA turnover suggest a very short half-life. In a study on fetal DNA in maternal plasma after delivery, Lo et al. (1999) estimated the mean half-life of circulating fetal DNA to be 16.3 min (range, 4–30 min). In another study evaluating the persistence of Epstein-Barr virus DNA in plasma after surgery for nasopharyngeal cancer, Chan et al. (2008) found a half-life of 139 min. Thus, the lag time of several months observed between the peak of AFB_1_ exposure and the peak of *R249S* serum levels in HBV carriers does not support that the release of *R249S* is the end product of mutagenesis and a rapid clearance of cells containing an *R249S* mutation. If this were the case, *R249S* DNA would occur in the bloodstream almost simultaneously to peak AFB_1_ exposure, a scenario seen in controls but not in HBV carriers. Thus, our results suggest that, in carriers, an interaction between AFB_1_ exposure and HBV might enhance the occurrence, persistence, and/or clonal expansion of cells with *R249S* mutations. In contrast, in controls, the formation and release of *R249S* were consistent with the seasonal pattern of exposure to AFB_1_, which suggests that the presence of *R249S* in the serum of subjects with no detectable neoplastic liver lesion may serve as a biomarker of mutagenesis following dietary aflatoxin exposure. *TP53* mutation load was associated with smoking dose and duration in a case–control study of lung cancer (Hagiwara et al. 2006), supporting the fact that mutations in CFDNA may serve as reporters of diverse forms of carcinogenic exposures.

In a previous case–control study of HCC in the Gambia (Lleonart et al. 2005), we showed that high plasma concentrations of *R249S* (> 10,000 copies/mL) were associated with HCC status (OR = 20; 95% CI: 5.6, 69.0). However, traces of *R249S* (0–2,500 copies) were found in many controls. In combination with our findings in the present study, results suggest that *R249S* in CFDNA may constitute a biomarker of exposure or a predictor of liver cancer, depending upon levels and temporal variation, such that low, transient plasma concentrations may reflect seasonal exposure to AFB_1_, whereas high and sustained plasma concentrations may indicate the presence of a developing cancer lesion. Prospective studies on chronic HBV carriers in China have shown that *R249S* concentrations could be detected before HCC diagnosis, with a tendency to increase in *R249S* proportion with decreasing time to diagnosis (Jackson et al. 2003; Szymañska et al. 2009). Further studies are needed to determine quantitative serum *R249S* thresholds as well as time-dependent variations that may distinguish subjects with ongoing AFB_1_ exposure from subjects with liver lesions progressing toward HCC.

## Conclusion

The presence and amount of *TP53 R249S* mutation in serum varied among survey subjects according to season, with a pattern that was distinct from the well-known seasonal variations in exposure to aflatoxin. Seasonal patterns differed between HBV carriers and noncarriers, suggesting a synergistic effect between HBV and aflatoxin exposure. This is the first demonstration of such an effect, in which being an HBV carrier appears to have a profound impact on the persistence of mutations induced by aflatoxin. This study also demonstrates that, in this particular exposure context, levels of *R249S* vary seasonally, suggesting that this mutation occurs multiple times in HBV carriers. A longitudinal study on HBV carriers exposed to aflatoxin is needed to determine how these variations influence the risk of developing chronic liver disease and, ultimately, HCC.

## Supplemental Material

(548 KB) PDF
