# Supplementary material for: Seasonal Variation in TP53 R249S-Mutated Serum DNA with Aflatoxin Exposure and Hepatitis B Virus Infection
Source: Environ Health Perspect. 2011 Jul 18;119(11):1635–40. doi: 10.1289/ehp.1103539 (PMC3226502; doi:10.1289/ehp.1103539)
Supplement: (548 KB) PDF [file ehp.1103539.s001.pdf]

Supplementary Material

Seasonal Variation in *TP53 R249S*-mutated Serum DNA With Aflatoxin Exposure And Hepatitis B Virus Infection

Stéphanie Villar, Emilie Le Roux-Goglin, Doriane A. Gouas, Amelie Plymoth, Gilles Ferro, Mathieu Boniol, Myriam Lereau, Ebrima Bah, Andrew J. Hall, Christopher P. Wild, Maimuna Mendy, Helene Norder, Marianne van der Sande, Hilton Whittle, Marlin D. Friesen, John D. Groopman, Pierre Hainaut.

A. GENOTYPE E

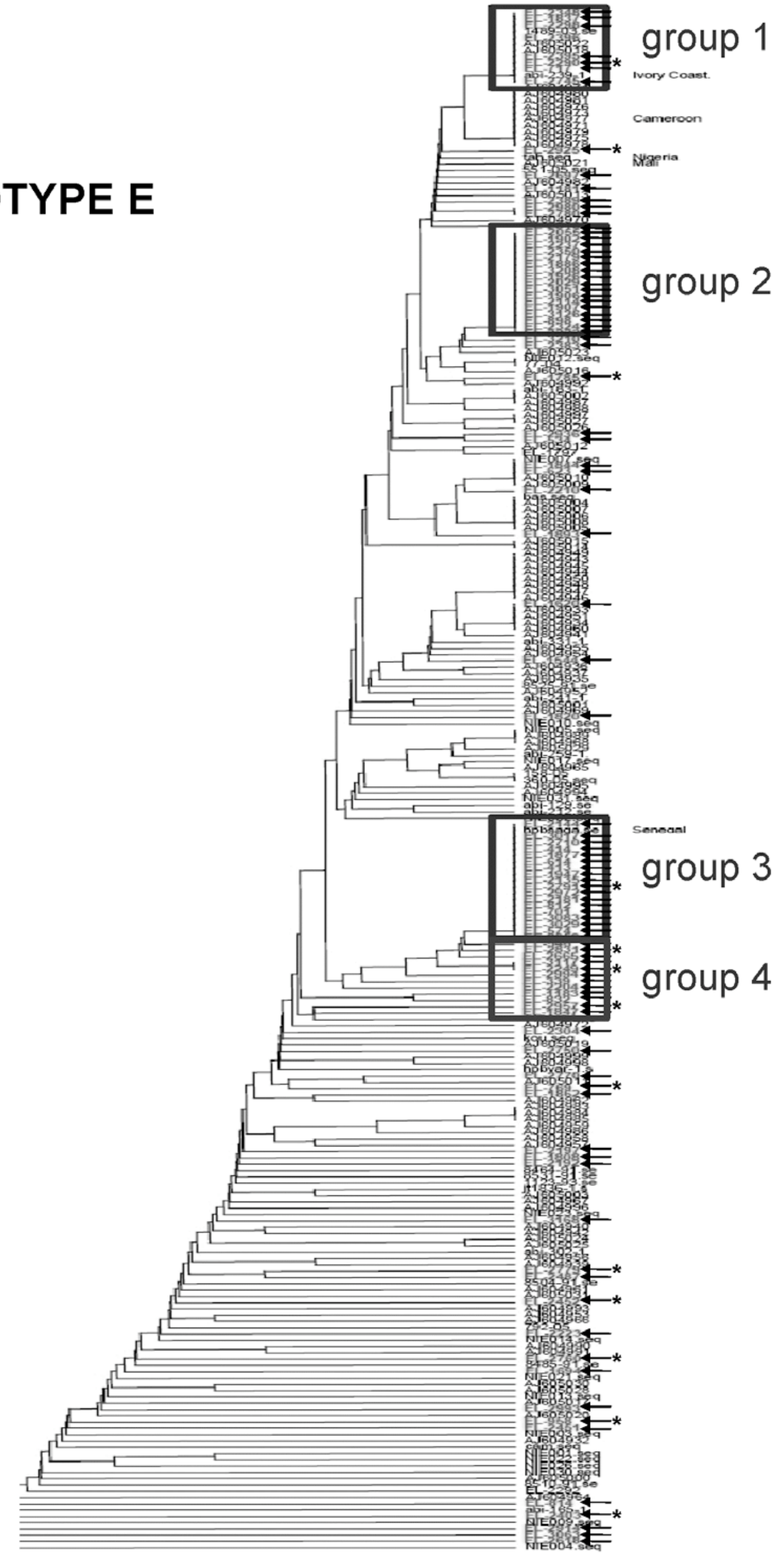
group 1
Ivory Coast.
Cameroon
Nigeria
Mali
group 2
Senegal
group 3
group 4

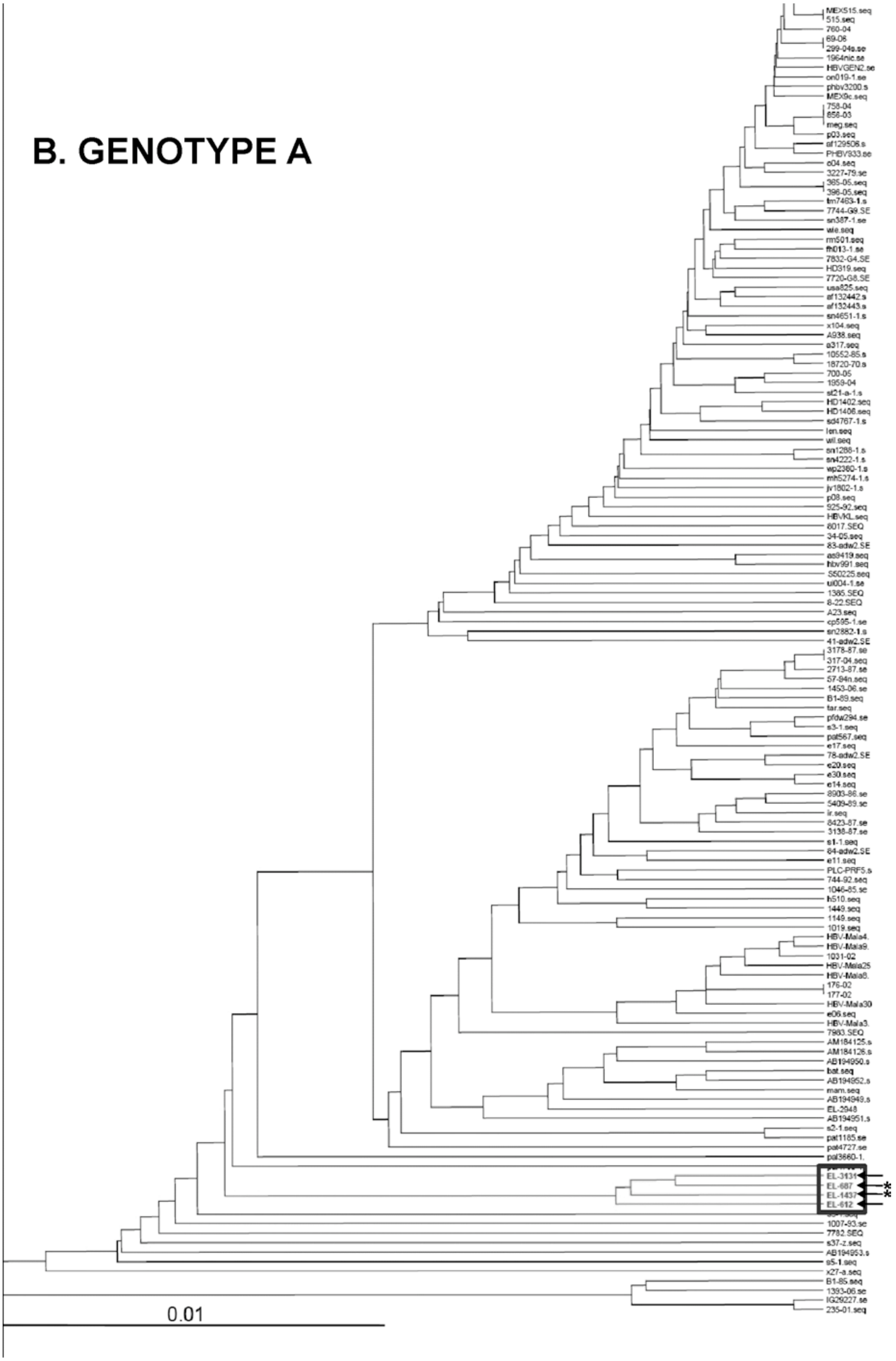
B. GENOTYPE A
0.01

Supplemental Material, Figure S1. Dendogram analysis using *HBS* gene. A: Among the 95 subjects from genotype E (arrows), 4 groups were isolated including HBV strains highly conserved (group 1-4) and 13 individuals were positive for HBV $1762^{T}/1764^{A}$ double mutation (arrows with stars). **B:** Among the 4 subjects from genotype A, 2 subjects were positive for HBV $1762^{T}/1764^{A}$ double mutation.
